# Heart Rate Variability May Predict the Severity of Appendicitis: A Cross-Sectional Study

**DOI:** 10.30476/BEAT.2022.91590.1282

**Published:** 2022-04

**Authors:** Alimohammad Bananzadeh, Abtin Vahidi, Sarvenaz Salahi, Ali Foroutan, Leila Ghahramani

**Affiliations:** 1 *Colorectal Research Center, Shiraz University of Medical Sciences, Shiraz, Iran*; 2 *Minimally Invasive Surgery Research Center, Iran University of Medical Sciences, Tehran, Iran*; 3 *Burn and Wound Healing Research Centre, Shiraz University of Medical Sciences, Shiraz, Iran*

**Keywords:** Appendicitis, Heart rate variability, Medication therapy management, Cross-sectional study

## Abstract

**Objective:**

To evaluate the role of heart rate variability (HRV) in predicting pre-operative severity of appendicitis.

**Methods:**

In this cross-sectional study, 171 cases of acute appendicitis who underwent appendectomy were enrolled. Pre-anesthetic pulse rate of included patients were documented while the severity of appendicitis was determined by intra-operative evidences reported by two independent surgeons. Demographic characteristics, laboratory variables, and Alvarado criteria were recorded.

**Results:**

The mean age of patients was 28.75±4.21 years; 54% were men. HRV negatively associated the severity of appendix inflammation. A positive association was found between HRV and omental wrapping and Alvarado score (*p*<0.01). The receiver operating characteristic (ROC) curve analysis demonstrated that HRV could differentiate simple and complicated appendicitis with a sensitivity of 78.5% and specificity of 97.2%.

**Conclusion:**

The present findings revealed that HRV may predict the pre-operative severity of appendicitis and help differentiate simple and complicated appendicitis.

## Introduction

Acute appendicitis is one the most common surgical emergencies with a lifetime risk of 7-8% [1]. It is categorized into simple and complex appendicitis based on the severity of inflammation [2]. Both simple or complicated appendicitis can be managed intrinsically by the omental wrap or the phlegmon formation [2]. Failure of omental wrapping around the perforated appendix can lead to the generalized peritonitis which increases the mortality rate [3]. A healthy immune system can localize the infection despite the perforation of the appendicitis [4].

The non-operative, conservative management of uncomplicated appendicitis has been recently suggested [5, 6]. This were consisted of antibiotic therapy without appendectomy [7, 8]. Thus, the pre-operative diagnosis of the appendicitis severity can help to predict the outcome of conservative management and more reasonable patient selection for non-operative treatment [9, 10]. It also helps to identify hospitalized patients for potential short surgery delays with minimal side effects [11-13]. However, a reliable discriminator of appendicitis severity for the selection of the patients is yet to be designed [14].

As a neural pathway, inflammatory reflex can control and down-regulate the inflammation through the activation of vagus nerve [15]. Westerloo *et al*., reported that the vagus nerve integrity is vital to decrease the inflammation during septic peritonitis [16]. Therefore, measurement of vagus nerve activity may estimate the state of inflammatory and anti-inflammatory balance. As the vagus nerve controls the heart rate, it has been shown that heart rate variability measurement can be considered a surrogate for evaluating the level of inflammation [17, 18]. Heart rate variability (HRV) is measured by the variation of pulse rate through the established intervals which can evaluate the balance between sympathetic and parasympathetic activity [19]. 

Given the above-mentioned issues, this study aimed to assess HRV, to predict the severity of the appendicitis before surgery, and also to compare HRV with other lab parameters such as white blood cells (WBC) and clinical scoring systems such as Alvarado score.

## Materials and Methods

*Study Design*

All adult patients diagnosed as acute appendicitis and scheduled for appendectomy from 2016 to 2017 were enrolled in this cross-sectional study. The present study was conducted in accordance with the principles of the revised *Declaration of Helsinki* and was approved by the local Ethics Committee of Shiraz University of Medical Sciences, Shiraz, Iran (IR.SUMS.REC.1398.040).

The inclusion criteria were patients with age >18 years who were scheduled for an appendectomy. Patients with prior history of cardiac diseases including arrhythmias, history of autoimmune disease, immunodeficiency, history of previous abdominal surgery, and history of using drugs affecting the cardiovascular or nervous system, including alpha or beta-blockers, vagomimetic or vagolytic drugs, anti-epileptic, and anti-depressant drugs were excluded. 

Acute appendicitis was diagnosed clinically (serial examination and Alvarado scoring system) in patients with Alvarado score of 4 to 6 confirmation was obtained by radiologic evidences, either ultrasound or computed tomography, or by serial exam convincing the surgeon for high clinical probability [20]. Radiologic confirmation was not needed in patients with an Alvarado score of 7 or more.

All eligible patients were included in the study by convenient sampling method. Demographic characteristics, comorbidities, laboratory data including white blood cells (WBC counts), and Alvarado criteria (migrating pain, anorexia, nausea/vomiting, tenderness, rebound tenderness, fever, leukocytosis, left shift in polymorphonuclear cells) were recorded. 

*Heart Rate Variability Recording*

Fifteen minutes prior to induction of anesthesia, patients’ heart rate was recorded using a heart rate monitoring probe. (Polar RS800, Polar Electro Oy, Kempele, Finland). Heart rate was recorded for 3 minutes. A co-ordination was performed with one surgeon for the operation to remove the bias of different surgeons’ skills. The surgeon was blinded about heart arte variability (HRV) outcomes. 

*Assessment of Intra-abdominal Inflammation*

An open appendectomy was performed by the double ligation method. Before the surgery and before appendectomy, another surgeon blinded to the HRV results would independently inspect and examine the abdomen and scored the intra-abdominal inflammation. The severity of appendicitis was classified into eight categories based on the gross and intraoperative findings recorded by surgeon: normal, mild and moderate inflammation, severe inflammation, suppurative, gangrenous, perforated, phlegmon, and omental wrapping. Appendicitis was considered complicated if gangrene, perforation, or abscess formation were found and simple if categorized as inflamed, suppurative, and phlegmon formation. At the end of the operation, all specimens were sent for histopathological evaluation.

*Statistical Analysis*

Descriptive results were reported as frequency (percentage) and mean±standard deviation (SD) for qualitative and quantitative variables, respectively. Kolmogorov–Smirnov test was used for evaluating the normal distribution of data. Independent sample *t *test or Mann Whitney U test was used to compare the quantitative variables among groups. On the other hand, categorical variables were compared using the chi-square test. The association of quantitative variables was tested by Pearson’s or Spearman’s correlation coefficient. The linear regression model was used to evaluate the effect of HRV on appendicitis severity, and the area under the curve (AUC) of receiver operating characteristic (ROC) curve was used to report the sensitivity and specificity of HRV to predict the complicated appendicitis. The statistical software of SPSS v.21 (IBM Corp. NY, USA) was used for the statistical analysis. *P* values of 0.05 or less were considered statistically significant.

## Results

*Demographic Characteristics and Alvarado Score*

One hundred seventy-one patients with acute appendicitis were enrolled according to post-op results. The mean age was 28.75±4.21 years; 54% of cases were men. Mean Alvarado score was 7.11±1.12. Twenty-six (16%) of cases had Alvarado scores 4-6, and 145 (84%) patients had Alvarado scores of seven more.

*Intra-operative Inflammation*

Twenty-four (14%) patients had mild to moderate inflammation, 75 (44%) had severe inflammation, 45 (26%) had suppurative appendicitis. Perforated appendicitis was found in 17 (10%) cases as well as 5 (3%) with gangrenous appendicitis, and 5 (3%) with phlegmon. The omental wrapping was positive in 29% (n=50) of patients. 

*Heart rate Variability*

As shown in Table 1, mean HRV in all patients was 70.18±6.27. There was a significant difference in mean HRV values based on the severity and type of appendicitis (*p*<0.001) with the highest value in patients with mild to moderate inflammation. Additionally, mean HRV was significantly higher in patients with omental wrapping than patients without omental wrapping (79.24±5.27 versus 66.50±11.38 (*p*<0.001)). 

**Table 1:** Heart rate variability based on the severity and type of appendicitis

	**Minimum **	**Maximum **	**Mean **	**Standard deviation**	**95% confidence interval**	
					**Upper Bound**	**Lower Bound**
Mild and moderate appendicitis	64.00	93.00	78.7500	6.01628	76.2095	81.2905
Severe appendicitis	58.00	90.00	76.8267	5.85033	75.4806	78.1727
Suppurative appendicitis	54.00	72.00	65.0455	5.04378	63.5120	66.5789
Gangrene appendicitis	45.00	60.00	52.4000	5.77062	45.2348	59.5652
Locally perforated appendicitis	40.00	52.00	46.8333	2.99509	45.3439	48.3228
Phlegmon	68.00	88.00	76.4000	7.53658	67.0421	85.7579
Total	40.00	93.00	70.1813	6.27924	68.4333	71.9293

Of all patients, 30% were complicated; mean HRV, WBC, Alvarado score, age and frequency of clinical findings were compared between patients with simple and complicated appendicitis in Table 2. The mean HRV was significantly higher in patients with simple appendicitis compared to the complicated ones (*p*<0.001), Other parameters including clinical findings, Alvarado score, WBC count and age was not significantly different between patients with simple and complicated appendicitis.

**Table 2:** Comparing mean heart rate variability, Alvarado score, white blood cells and frequency of clinical findings between patients with simple and complicated appendicitis

	**Simple**	**Complicated **	***p*** ** value**
Heart rate variability, mean (SD)	73.52 (8.00)	53.10 (12.07)	<0.001^a^
White blood cell (×10^9^/L), mean(SD)	13.13 (1.68)	16.52 (3.19)	0.201^a^
Alvarado score, mean (SD)	7.25 (0.818)	7.96 (0.881)	0.472^a^
Age, mean (SD)	27.825 (8.46)	32.857 (12.52)	0.205^a^
Anorexia	78.0%	82.0%	0.381^b^
Nausea/vomiting	82.2%	79.1%	0.310^b^
Pain shift	88.0%	100%	0.198^b^
Tenderness	92.0%	100%	0.247^b^
Rebound	89.0%	100%	0.357^b^
Fever	76.2%	91.1%	0.184^b^

^a^The results of independent samples *t* test;^ b^The results of chi square test.

In the next step, logistic regression (Forward LR) was used to investigate the association of age, sex, HRV, WBC, and Alvarado score with simple/complicated appendicitis. The results showed that only HRV was associated with complicated appendicitis (OR: 0.869; *p*<0.001). In contrast, age, sex, WBC count and Alvarado score were not significantly associated with complicated appendicitis (Table 3).

**Table 3:** Logistic regression analysis for association of Sex, Age, white blood cells, Alvarado score and heart rate variability with Simple versus complicated appendicitis

	**B**	**S.E.**	**Wald**	**df**	**Exp(B)**	***p *** **value**
HRV	-0.140	0.034	16.823	1	0.869	<0.001
Sex						0.761
Age						0.249
WBC						0.232
Alvarado score						0.151

**Fig. 1:**
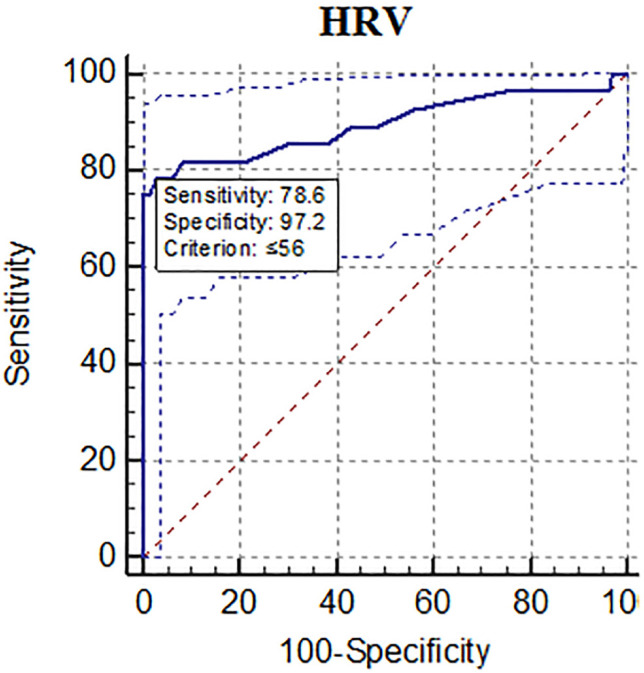
The receiver operating characteristic (ROC) curve for the ability of heart rate variability

To study the ability of HRV for prediction of complicated appendicitis, we used the ROC curve, the results of which are shown in Figures 1. As shown, HRV had an AUC of 0.895, revealed a sensitivity of 78.57% and specificity of 97.20% at cut-off the level of ≤56.

## Discussion

In this study, we examined the association between HRV with several variables to indicate the predictive value of HRV in appendicitis severity. One of the important variables was the Alvarado score, which was negatively correlated with HRV. In the same way, HRV was the only variable which associated with complicated appendicitis, while WBC and Alvarado score did not have any correlation with severe cases of appendicitis. However, a significant negative correlation was observed between WBC count and HRV.

In our study, most of the patients were men and young with Alvarado scores of seven and more. Severe inflammation was found in near half of the patients. As shown, HRV could differentiate based on the severity of appendicitis, as highest level of HRV was found in cases of mild to moderate appendicitis. Additionally, mean HRV was significantly higher in patients with omental wrapping than patients without omental wrapping. After assessment of the role of WBC percentage and HRV for prediction of appendicitis severity, WBC percentage had the cut-off more than 15300 per microliter and HRV with the cut-off lower than 56 had the highest sensitivity and specificity, respectively. 

In one review study on 42 studies, which indicated score >7 as the appropriate cut–off for diagnosis of acute appendicitis and surgery [21], the higher Alvarado score was associated with lower HRV, and evaluation of the association between Alvarado criteria with HRV showed WBC count as the only item related to HRV. This result confirmed our study’s initial hypothesis that HRV increased as a defensive mechanism to control the appendicitis-related inflammation. Therefore, the patients with a lower HRV had a higher level of inflammation, and higher HRV can control inflammation, which confirmed the present study results. We examined the intraoperative findings despite the clinical examination based on the Alvarado scoring system, and the results showed that the higher severity of appendicitis was associated with reduced HRV. We also looked omental wrapping, considered a defensive immunologic mechanism of omentum to control inflammation and microbes [22]. The results showed higher mean of HRV in patients with omental wrapping. These results were confirmed by previous investigations about the role of HRV in controlling inflammation [23]. 

Studying the association between HRV and the status of diseases has revealed that HRV can be used as a predictive and prognostic factor in several clinical conditions associated with the autonomic nervous system including cardiovascular diseases, diabetes, and hypertension [24]. In contrast, it has not been studied in appendicitis. Our results showed that HRV ≤56 had a sensitivity of 78.57% and specificity of 97.20%. These results indicate that HRV can be used as an efficient and available diagnostic tool for complicated appendicitis. Despite the assessment of HRV in the chronic diseases [24, 25], few studies have investigated the diagnostic accuracy of HRV for acute conditions. 

Studies have clearly defined the role of HRV on risk assessment of patients with acute myocardial infarction (AMI) [25-27]. Ahmad *et al*., investigated the risk of sepsis in 17 patients undergoing bone marrow transplant. They reported that continuous HRV monitoring (up to 16 days) in ambulatory patients could be an appropriate diagnostic factor of sepsis, and HRV drop <25% can alert the physician 24 hours before the presentation of clinical symptoms, which enables early diagnosis and treatment of sepsis [28]. Other researchers have also confirmed the role of HRV for sepsis prediction in adults and neonates [29, 30]. Although the results of these studies confirmed the present association of HRV with inflammation, differences in the type of disease necessitated further studies on the role of HRV in appendicitis.

This study was one the few reports about the role of HRV to predict the severity of appendicitis. Which could help as a discriminator for the patient selection of appendicitis non operative management. In this study, we found that HRV had a negative association with severity of appendicitis and could predict and detect the complicated cases of appendicitis. Therefore, HRV could be used for prioritization of patients for appendectomy. Though, heart rate (HR) must be checked for all patients with appendicitis and the measuring tool of this parameter have been available in outpatient and inpatient settings, the variability in HR could help us to prioritize patients persistently in short and long time intervals.

In conclusion, the present study results revealed that HRV was negatively related with Alvarado score, WBC count, and intraoperative parameters including the severity of inflammation and omental wrapping. Also, studying the diagnostic accuracy of WBC and HRV showed that not only WBC and its combination with HRV, but also HRV alone could detect the complicated appendicitis cases with high sensitivity and specificity. Therefore, we suggest HRV as an easily accessible tool for preoperative assessment of appendicitis severity and further management planning. Further studies are required to prove this tool’s accuracy and clinical applicability.

Nevertheless, the cross-sectional nature of the study limited the evaluation of causative relationships between HRV and appendicitis. Furthermore, we did not follow patients after discharge and could not evaluate patients’ long–term outcomes. Another limitation of the present study was that we only assessed HRV before induction of anesthesia in patients scheduled for surgery and estimated the diagnostic accuracy of HRV based on statistical analysis. On the other hand, the clinical applicability of HRV assessment for the diagnosis of complicated appendicitis has to be clinically confirmed possibly by running clinical trials.

## Declarations

### Ethics approval and consent to participate:

This study was supported by Shiraz University of Medical Sciences (SUMS) (IR.SUMS.Rec.1398.040).

### Consent for publication:

All authors agree with the publication of this article.

### Conflict of Interests:

The authors declared that they have no conflict of interest.

### Funding:

This study was supported by Shiraz University of Medical Sciences (SUMS).

### Authorship contribution statement:

A. Bananzadeh: Conceptualization, Methodology; A. Vahidi: Writing, original draft, Investigation, formal analysis, visualization; S. Salahi: Writing, review and editing; A. Foroutan: Methodology, writing, review and editing; L. Ghahramani: Writing, review and editing.

### Acknowledgement:

None declared.
